# Predictive Factors and the Predictive Scoring System for Falls in Acute Care Inpatients: Retrospective Cohort Study

**DOI:** 10.2196/58073

**Published:** 2025-01-13

**Authors:** Chihiro Saito, Eiji Nakatani, Hatoko Sasaki, Naoko E Katsuki, Masaki Tago, Kiyoshi Harada

**Affiliations:** 1 Department of Nursing, Shizuoka General Hospital, Shizuoka, Japan; 2 Graduate School of Public Health, Shizuoka Graduate University of Public Health, 4-27-2, Kita-ando, Aoi-ku, Shizuoka, 420-0881, Japan, 81 54-295-5400, 81 54-248-3520; 3 Research Support Center, Shizuoka General Hospital, Shizuoka, Japan; 4 Department of Biostatistics and Health Data Science, Graduate School of Medical Science, Nagoya City University, Nagoya, Japan; 5 Department of General Medicine, Saga University Hospital, Saga, Japan; 6 Department of Medical Safety, Shizuoka General Hospital, Shizuoka, Japan

**Keywords:** falls, inpatient falls, acute care hospital, predictive factor, risk factors

## Abstract

**Background:**

Falls in hospitalized patients are a serious problem, resulting in physical injury, secondary complications, impaired activities of daily living, prolonged hospital stays, and increased medical costs. Establishing a fall prediction scoring system to identify patients most likely to fall can help prevent falls among hospitalized patients.

**Objectives:**

This study aimed to identify predictive factors of falls in acute care hospital patients, develop a scoring system, and evaluate its validity.

**Methods:**

This single-center, retrospective cohort study involved patients aged 20 years or older admitted to Shizuoka General Hospital between April 2019 and September 2020. Demographic data, candidate predictors at admission, and fall occurrence reports were collected from medical records. The outcome was the time from admission to a fall requiring medical resources. Two-thirds of cases were randomly selected as the training set for analysis, and univariable and multivariable Cox regression analyses were used to identify factors affecting fall risk. We scored the fall risk based on the estimated hazard ratios (HRs) and constructed a fall prediction scoring system. The remaining one-third of cases was used as the test set to evaluate the predictive performance of the new scoring system.

**Results:**

A total of 13,725 individuals were included. During the study period, 2.4% (326/13,725) of patients experienced a fall. In the training dataset (n=9150), Cox regression analysis identified sex (male: HR 1.60, 95% CI 1.21‐2.13), age (65 to <80 years: HR 2.26, 95% CI 1.48‐3.44; ≥80 years: HR 2.50, 95% CI 1.60‐3.92 vs 20-<65 years), BMI (18.5 to <25 kg/m²: HR 1.36, 95% CI 0.94‐1.97; <18.5 kg/m²: HR 1.57, 95% CI 1.01‐2.44 vs ≥25 kg/m²), independence degree of daily living for older adults with disabilities (bedriddenness rank A: HR 1.81, 95% CI 1.26‐2.60; rank B: HR 2.03, 95% CI 1.31‐3.14; rank C: HR 1.23, 95% CI 0.83‐1.83 vs rank J), department (internal medicine: HR 1.23, 95% CI 0.92‐1.64; emergency department: HR 1.81, 95% CI 1.26‐2.60 vs department of surgery), and history of falls within 1 year (yes: HR 1.66, 95% CI 1.21‐2.27) as predictors of falls. Using these factors, we developed a fall prediction scoring system categorizing patients into 3 risk groups: low risk (0-4 points), intermediate risk (5-9 points), and high risk (10-15 points). The c-index indicating predictive performance in the test set (n=4575) was 0.733 (95% CI 0.684‐0.782).

**Conclusions:**

We developed a new fall prediction scoring system for patients admitted to acute care hospitals by identifying predictors of falls in Japan. This system may be useful for preventive interventions in patient populations with a high likelihood of falling in acute care settings.

## Introduction

In 2022, the proportion of the population aged 65 years or older in Japan reached a record high of 29% [1]. A 2017 survey by the Ministry of Health, Labour and Welfare of Japan revealed that patients aged 75 years or older accounted for 41.5% of all patients admitted to acute care hospitals [2]. Given the trend in population aging, it is projected that the number of older adult patients with a high risk of falling will further escalate in the future. Falls are not limited to older adults, and falls in hospitalized patients can lead to severe physical injuries, secondary complications, a marked decline in activities of daily living (ADL), and even death in extreme cases [3,4]. Therefore, fall prevention has become an important issue to protect patients’ lives and quality of life [5].

Interventions for fall prevention must be strategically targeted to populations with a high risk of falling during hospitalization. Furthermore, previous studies have emphasized the importance of patient exercise therapy [6,7] and education for both patients and health care providers in fall prevention [8-12]. Educating patients about the risks of falls and strategies to mitigate these risks is crucial in reducing the incidence of falls in hospitalized patients. To effectively conduct patient education, it is imperative to construct a fall prediction model for the accurate identification of these high-risk patients. Currently, fall prevention measures in hospitals include fall prediction models using information from electronic health record (EHR) systems [13-22], as well as predictive models that analyze patient information from EHRs and nursing records using artificial intelligence [15,23]. Here, we present several fall prediction models that can be used with EHRs [17-20]. The STRATIFY scale [17] uses a history of falls, visual impairment, mental status, frequency of elimination, and ability to transfer and move as factors in a prediction model. The Morse Fall Scale [18] includes 6 items related to a history of falls, comorbidities, use of walking aids, intravenous fluids, ability to walk and move, and mental status. The Medication Fall Risk Score and Evaluation Tool [19] assesses the medication-related fall risk. This tool considers a patient’s use of medications as predictors, classified according to the associated risk levels. Tago et al [20] reported 8 predictors of falls in people with disabilities in Japan: age, sex, emergency hospitalization, admission to neurosurgery, use of sleeping pills, history of falls, independence in eating, and level of independence in daily living.

Although fall prediction tools are widely used in Japanese hospitals, existing models vary in predictors and are often difficult to apply due to differences in facility and patient characteristics. Many hospitals also rely on tools that lack a strong evidence base [24,25] and are not well integrated with EHR systems. In our clinical environment, we found it challenging to implement existing models due to their reliance on numerous variables that are either difficult to extract from the EHR or unavailable. Therefore, we aimed to develop a streamlined fall risk assessment tool that considers facility-specific factors and can be seamlessly integrated with EHR systems, enabling real-time insights and more efficient fall prevention strategies.

The purpose of this study was to identify predictive factors for falls in patients admitted to an acute care hospital as well as to develop a scoring system using these factors and evaluate its validity.

## Methods

### Participants and Study Design

**Figure 1.**
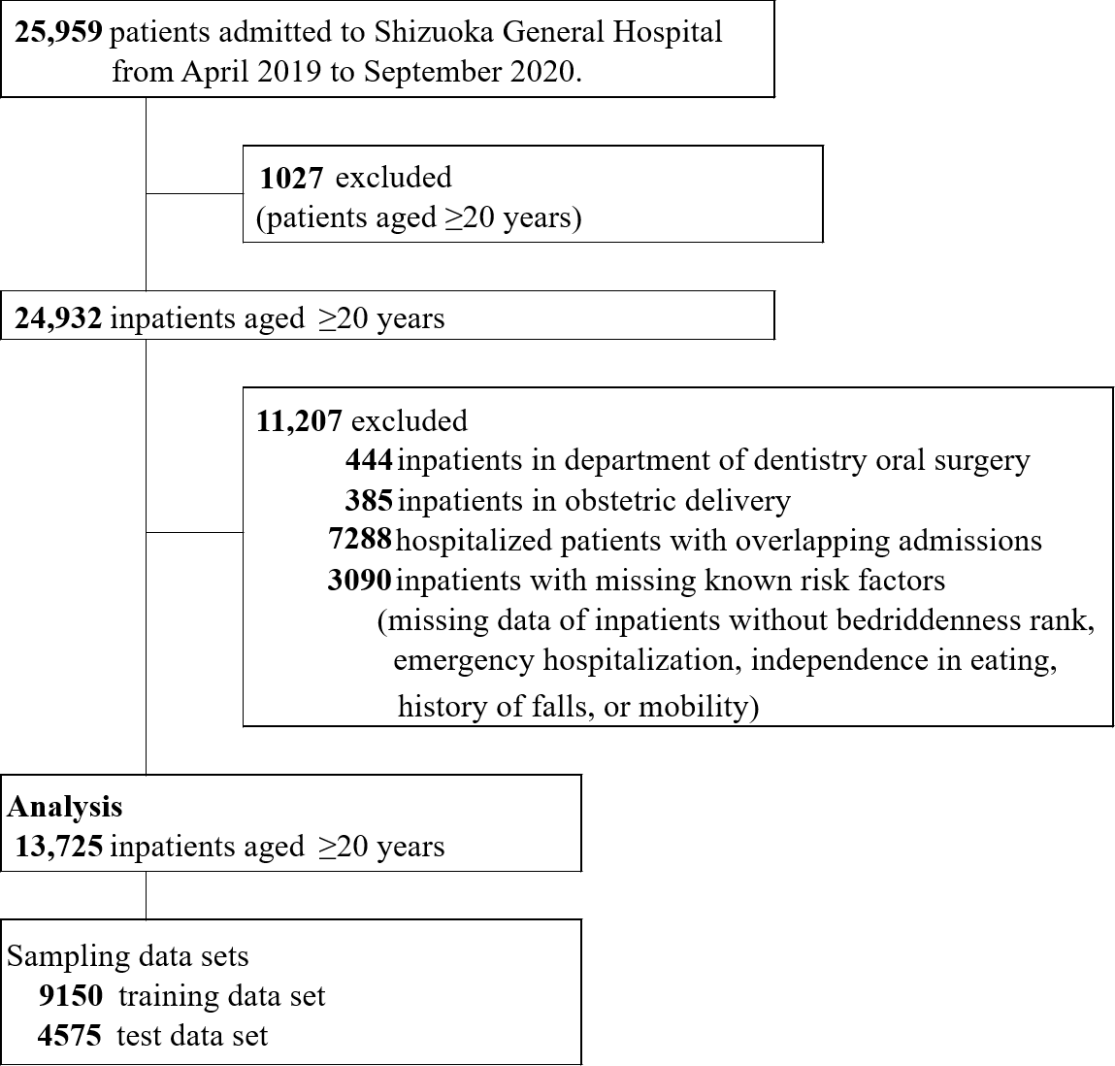
Flowchart illustrating the study population.

We conducted a retrospective cohort study of patients aged ≥20 years admitted to Shizuoka General Hospital between April 2019 and September 2020. Inpatients excluded from the study included those not covered by the Diagnosis Procedure Combination system, such as dental and oral surgery patients and obstetrics and gynecology inpatients during pregnancy, childbirth, and postpartum. In addition, inpatients lacking data on known risk factors such as the degree of independence in daily living for the older adults with disabilities, bedriddenness rank (BR), emergency admission, dietary independence, mobility, and history of falls in the past year were also excluded, as illustrated in Figure 1.

### Variables at Hospitalization as Candidate Predictive Factors

All data used in this study were extracted from patients’ medical records as of February 3, 2022. Preadmission medical history variables included dementia [5,6], Parkinson disease [7,26], stroke [6], visual impairment (with or with no diagnosis of glaucoma or cataracts) [4,5], history of falls [5,16,20,27], and use of sleep medications [20,28-30]. The following variables at admission were also collected: age [6,16,20,27], sex [20,27,28], BMI [31-35], date of admission [20], disease name at admission, department [20], mode of admission, ambulance transport, consciousness disorders [16,23,28], requirement for nursing care, good sleep condition, use of sleeping medication [20,28,30], status of medication management, BR [20,36], Cognitive Function Scores [5,6], ADL at admission (eating, transferring, dressing, toilet transfer or use, bathing, level walking, stair use, changing clothes, defecation management, and urinary management) [17,18,20], fall assessment end points at admission (history of falls or falls within 1 year, inability to stand without holding on [28], impaired judgment and comprehension, toilet assistance, and use of portable toilet), and presence of physical restraint screening at admission.

The BR [20,36] is an official assessment tool in Japan’s long-term care insurance system [37]. The BR is an assessment of the degree to which a person’s daily life is restricted; this degree is mainly assessed in terms of mobility in daily life, such as whether the person is independent, in a wheelchair, or bed bound. The Ministry of Health, Labour and Welfare ranks the degree of BR based on evaluations by nurses and other health care professionals according to the daily care they provide during hospitalization, as well as on reports from family members. The procedure for assessing BR and its reliability have been reported [20,36].

### Falls During Hospitalization as an Outcome

The primary end point was the time from the date of admission to a fall at incident level [38] 2 or higher (hereafter referred to as “fall”), which requires medical resources. For patients who died during hospitalization and those who did not have a fall, the date of death or date of discharge, respectively, was used as the censoring date. The classification of incident levels is shown in Table S1 in Multimedia Appendix 1.

### Statistical Analysis

Demographic data and potential predictors at the time of hospital admission were summarized as follows. Continuous variables are described using mean (SD) or median (range), considering the distribution type. Categorical variables are summarized as frequency (%). For comparisons between the groups with and with no fall occurrence, *t* tests were used for continuous variables and chi-squared tests for categorical variables. The Kaplan-Meier method was applied to estimate the fall rate.

We explored predictors of falls and constructed a predictive model using two-thirds of the total cases, randomly selected as the training group, with the remaining one-third serving as the validation group for the scoring system. In the training group, predictive factors were identified using the Cox proportional hazards model, and we calculated hazard ratios (HRs), 95% confidence intervals (CIs), and *P* values. Predictive candidates that were significant (*P*<.05) in the comparison of backgrounds among patients with and with no falls, along with known predictors, were included in a multivariable model. Factors with *P* value of <.2 in this model were identified as predictors of inhospital falls. Independence between explanatory variables was confirmed using an absolute value of Spearman rank correlation coefficient of >0.4. Among 2 correlated variables, 1 was chosen based on ease of collection or clinical significance; this variable was then included in the multivariable model. According to the identified predictive factors, a score was created for each HR, and these scores were summed. We used a method called “conditional inference tree analysis” to categorize patients into 3 groups based on their risk of falling. This approach works by first dividing the data into 2 groups based on their overall scores. Then, a statistical test is performed to see whether these 2 groups are significantly different, and the variable that shows the strongest difference (the one with the lowest *P* value) is used to split the groups. This process is repeated within each subgroup until no further meaningful divisions can be made or the smallest group size allowed is reached. The predictive performance of this score and the fall risk groups in the validation group were evaluated using the c-index.

The significance level of the 2-tailed test was set at .05. Missing values were not imputed in the analyses. All analyses were performed using R (version 4.1.1; The R Foundation for Statistical Computing), EZR (version 1.54; Saitama Medical Center, Jichi Medical University) [39], and IBM SPSS (version 28; IBM Corp).

### Ethical Considerations

This study conformed to the Ethical Principles for Medical Research Involving Human Subjects issued by the Ministry of Health, Labour and Welfare and the Ministry of Education, Culture, Sports, Science and Technology of Japan. Following these guidelines, the Shizuoka General Hospital research ethical committee determined that individual patient informed consent was not required because we analyzed existing information in this study, and patients were given the right to refuse participation via disclosure. After obtaining committee approval (SGHIRB #2020075; January 15, 2022) and publishing the disclosure document on Shizuoka General Hospital’s website, the information for each individual was anonymized, and the analysis was conducted.

## Results

### Patient Background and Falls at Incident Level 2 or Higher on Admission

From April 1, 2019, to September 30, 2020, a total of 24,932 inpatients aged 20 years or older were admitted to Shizuoka General Hospital. We excluded 3.3% (829/24,932) of patients not covered by the Diagnosis Procedure Combination, 29.2% (7288/24,932) with duplicate admissions, and 12.4% (3090/24,932) with missing known risk factors of falls. Consequently, 55% (13,725/24,932) of patients were included in the analysis (Figure 1).

During the observation period, defined as the length of hospital stay (median [range]: 13 [1-271] days), 3.6% (489/13,725) of patients experienced falls across all incident levels, of which 2.4% (326/13,725) falls were classified as incident level 2 or higher. For this study, we used fall data meticulously managed by the hospital’s medical safety department, ensuring accuracy and reliability. The details are shown in Table S2 in Multimedia Appendix 1. The median age (range) of the patients included in the analysis was 66 (20-103) years, with 52.1% (7150/13,725) male patients, and the median BMI (range) was 22.8 (9.6‐58.1) kg/m^2^. Table S3 in Multimedia Appendix 1 shows the results of the comparison between the backgrounds of patients in the training and test datasets. In a univariable analysis of the training dataset (n=9150; two-thirds of the study population), we compared patient backgrounds according to the presence of falls (Table 1).

**Table 1.** Patient background on admission^a^.

Variable		With no fall (n=8934)	With fall (n=216)	*P* value
**Age**				<.001
	Age (years), mean (SD)	65.9 (17)	75.0 (11.5)	
**Age**				<.001
	20 to <65 years, n (%)	3347 (37.5)	28 (13)	
	65 to <80 years, n (%)	3673 (41.1)	105 (48.6)	
	≥80 years, n (%)	1914 (21.4)	83 (38.4)	
**Sex, n (%)**				<.001
	Female	4287 (48)	75 (34.7)	
	Male	4647 (52)	141 (65.3)	
**BMI**				<.001
	BMI, mean (SD)	22.9 (4.2)	21.1 (3.9)	
**BMI**				<.001
	<18.5 kg/m^2^, n (%)	1142 (12.8)	48 (22.2)	
	18.5 to <25 kg/m^2^, n (%)	5363 (60)	132 (61.1)	
	≥25 kg/m^2^, n (%)	2403 (26.9)	36 (16.7)	
	Missing, n (%)	26 (0.3)	0 (0)	
**Dementia, n (%)**				.927
	No	8697 (97.3)	211 (97.7)	
	Yes	237 (2.7)	5 (2.3)	
**Parkinson disease, n (%)**				.592
	No	8861 (99.2)	213 (98.6)	
	Yes	73 (0.8)	3 (1.4)	
**Stroke, n (%)**				.003
	No	8121 (90.9)	183 (84.7)	
	Yes	813 (9.1)	33 (15.3)	
**Visual impairment, n (%)**				.778
	No	7991 (89.4)	195 (90.3)	
	Yes	943 (10.6)	21 (9.7)	
**Cognitive function score, n (%)**				<.001
	No	7855 (87.9)	179 (82.9)	
	Yes	710 (7.9)	37 (17.1)	
	Missing	369 (4.1)	0 (0)	
**Ambulance transport, n (%)**				<.001
	No	7432 (83.2)	156 (72.2)	
	Yes	1502 (16.8)	60 (27.8)	
**Emergency admission, n (%)**				<.001
	Scheduled hospitalization	5528 (61.9)	74 (34.3)	
	Emergency hospitalization	3406 (38.1)	142 (65.7)	
**Department, n (%)**				.007
	Internal medicine	4496 (50.3)	118 (54.6)	
	Department of surgery	4025 (45.1)	80 (37)	
	Emergency department	413 (4.6)	18 (8.3)	
**Consciousness disorders, n (%)**				<.001
	No	8022 (89.8)	167 (77.3)	
	Yes	912 (10.2)	49 (22.7)	
**Bedriddenness rank, n (%)**				<.001
	Rank J	5500 (61.6)	65 (30.1)	
	Rank A	1568 (17.6)	58 (26.9)	
	Rank B	526 (5.9)	34 (15.7)	
	Rank C	1340 (15)	59 (27.3)	
**Eating, n (%)**				<.001
	Independent	7067 (79.1)	107 (49.5)	
	Requiring assistance	1867 (20.9)	109 (50.5)	
**Transferring, n (%)**				<.001
	Independent	6579 (73.6)	89 (41.2)	
	Requiring assistance	2355 (26.4)	127 (58.8)	
**Dressing, n (%)**				<.001
	Independent	7065 (79.1)	112 (51.9)	
	Requiring assistance	1853 (20.7)	104 (48.1)	
	Missing	16 (0.2)	0 (0)	
**Toilet transfer or use, n (%)**				<.001
	Independent	6820 (76.3)	94 (43.5)	
	Requiring assistance	2099 (23.5)	121 (56)	
	Missing	15 (0.2)	1 (0.5)	
**Bathing, n (%)**				<.001
	Independent	6673 (74.7)	95 (44)	
	Requiring assistance	2042 (22.9)	114 (52.8)	
	Missing	219 (2.5)	7 (3.2)	
**Level walking, n (%)**				<.001
	Independent	6707 (75.1)	93 (43.1)	
	Requiring assistance	2107 (23.6)	122 (56.5)	
	Missing	120 (1.3)	1 (0.5)	
**Stair use, n (%)**				<.001
	Independent	6541 (73.2)	86 (39.8)	
	Requiring assistance	1986 (22.2)	111 (51.4)	
	Missing	407 (4.6)	19 (8.8)	
**Changing clothes, n (%)**				<.001
	Independent	6799 (76.1)	97 (44.9)	
	Requiring assistance	2116 (23.7)	119 (55.1)	
	Missing	19 (0.2)	0 (0)	
**Defecation management, n (%)**				<.001
	Independent	7254 (81.2)	127 (58.8)	
	Requiring assistance	1618 (18.1)	87 (40.3)	
	Missing	62 (0.7)	2 (0.9)	
**Urination management, n (%)**				<.001
	Independent	7243 (81.1)	126 (58.3)	
	Requiring assistance	1625 (18.2)	88 (40.7)	
	Missing	66 (0.7)	2 (0.9)	
**History of falls within 1 year, n (%)**				<.001
	No	7830 (87.6)	153 (70.8)	
	Yes	1104 (12.4)	63 (29.2)	
**Inability to stand without holding, n (%)**				<.001
	No	5318 (59.5)	53 (24.5)	
	Yes	3486 (39)	159 (73.6)	
	Missing	130 (1.5)	4 (1.9)	
**Impaired judgment and comprehension, n (%)**				<.001
	No	7592 (85)	157 (72.7)	
	Yes	1116 (12.5)	53 (24.5)	
	Missing	226 (2.5)	6 (2.8)	
**Toileting assistance, n (%)**				<.001
	No	6694 (74.9)	106 (49.1)	
	Yes	1795 (20.1)	99 (45.8)	
	Missing	445 (5)	11 (5.1)	

a Between-group comparisons were made using *t* tests and *χ*2 tests for continuous and categorical variables, respectively. The *P* value was calculated using the Wald test. Bedriddenness rank J=independent or autonomous, rank A=housebound, rank B=chair, and rank C=bedridden.

### Predictors of Falls

In the training dataset, univariable Cox regression analysis compared patient backgrounds based on the presence or absence of falls and identified factors affecting the time from admission to the date of fall (Table 2, left side). Correlations between explanatory variables were checked using Spearman correlation coefficients, and independent explanatory variables were entered into a multivariable regression model. The variables were narrowed down by applying a high absolute value of the Spearman correlation coefficient (>0.4) (Table S4 in Multimedia Appendix 1). Among the 21 variables that were significant in the univariable analysis, 5 variables (age, sex, BMI, department, and history of falls within 1 year) were not correlated with each other. Sixteen variables (including 12 ADL-related variables, emergency admission, and consciousness disorders) had a correlation coefficient of 0.4 or higher. From the correlated variables, BR was ultimately chosen. This decision was influenced by the fact that 12 other ADL variables showed correlation, and BR could potentially explain physical severity, including factors such as emergency transport and impaired consciousness. The variables were chosen based on ease of collection or clinical significance.

**Table 2.** Univariable and multivariable Cox regression analysis results for fall rates in the training dataset^a^.

Variable (reference) and category		Training dataset (n=9150)					
		Univariable model			Multivariable model		
		HR^b^	95% CI	*P* value	HR	95% CI	*P* value
**Age**							
	1 year	1.03	1.02‐1.04	<.001	—^c^	—	—
**Age (20 to <65 years)**							
	65 to <80 years	2.71	1.79‐4.12	<.001	2.26	1.48‐3.44	<.001
	≥80 years	3.53	2.29‐5.43	<.001	2.50	1.60‐3.92	—
**Sex (female)**							
	Male	1.60	1.21‐2.12	.001	1.60	1.21‐2.13	.001
**BMI**							
	1 kg/m^2^	0.92	0.89‐0.96	<.001	—	—	—
**BMI (≥25 kg/m** ^**2**^ **)**							
	18.5 to <25 kg/m^2^	1.57	1.09‐2.27	.017	1.36	0.94‐1.97	.127
	≤18.5 kg/m^2^	2.01	1.30‐3.10	.002	1.57	1.01‐2.44	—
**Ambulance transport (no)**							
	Yes	1.77	1.33‐2.36	<.001	—	—	—
**Emergency admission (scheduled hospitalization)**							
	Emergency hospitalization	1.22	0.90‐1.64	.203	—	—	—
**Stroke (no)**							
	Yes	1.18	0.81‐1.72	.376	—	—	—
**Bedriddenness rank (rank J)**							
	Rank A	2.27	1.59‐3.23	<.001	1.81	1.26‐2.60	.001
	Rank B	2.93	1.93‐4.45	<.001	2.03	1.31‐3.14	—
	Rank C	1.94	1.35‐2.77	<.001	1.23	0.83‐1.83	—
**Cognitive function score (no)**							
	Yes	1.40	0.98‐2.01	.063	—	—	—
**Department (department of surgery)**							
	Internal medicine	1.36	1.02‐1.81	.034	1.23	0.92‐1.64	.119
	Emergency department	2.16	1.29‐3.60	.003	1.81	1.26‐2.60	—
**Consciousness disorders (no)**							
	Yes	1.47	1.06‐2.03	.020	—	—	—
**Eating (independent)**							
	Requiring assistance	2.05	1.56‐2.69	<.001	—	—	—
**Transferring (independent)**							
	Requiring assistance	2.12	1.60‐2.80	<.001	—	—	—
**Dressing (independent)**							
	Requiring assistance	1.88	1.43‐2.48	<.001	—	—	—
**Toilet transfer or use (independent)**							
	Requiring assistance	2.22	1.68‐2.93	<.001	—	—	—
**Bathing (independent)**							
	Requiring assistance	2.10	1.59‐2.78	<.001	—	—	—
**Level walking (independent)**							
	Requiring assistance	2.18	1.65‐2.88	<.001	—	—	—
**Stair use (independent)**							
	Requiring assistance	2.17	1.63‐2.90	<.001	—	—	—
**Changing clothes (independent)**							
	Requiring assistance	2.10	1.60‐2.77	<.001	—	—	—
**Defecation management (independent)**							
	Requiring assistance	1.63	1.23‐2.15	.001	—	—	—
**Urination management (independent)**							
	Requiring assistance	1.64	1.24‐2.17	.001	—	—	—
**History of falls within 1 year (no)**							
	Yes	2.01	1.50‐2.70	<.001	1.66	1.21‐2.27	.002
**Inability to stand without holding (no)**							
	Yes	2.51	1.83‐3.45	<.001	—	—	—
**Impaired judgment and comprehension (no)**							
	Yes	1.40	1.03‐1.92	.035	—	—	—
**Toileting assistance (no)**							
	Yes	2.10	1.59‐2.77	<.001	—	—	—

a Between-group comparisons were made using *t* tests and *χ*2 tests for continuous and categorical variables, respectively. The *P* value was calculated using the Wald test. Bedriddenness rank J= independent or autonomous, rank A=housebound, rank B=chair, and rank C= bedridden.

b HR: hazard ratio.

c Not applicable.

In multivariable analysis, age (65 to <80 years: HR 2.26, 95% CI 1.48‐3.44; ≥80 years: HR 2.50, 95% CI 1.60‐3.92 vs 20 to <65 years), sex (male: HR 1.60, 95% CI 1.21‐2.13), BMI (18.5 to <25 kg/m²: HR 1.36, 95% CI 0.94‐1.97; <18.5 kg/m²: HR 1.57, 95% CI 1.01‐2.44 vs ≥25 kg/m²), BR (rank A: HR 1.81, 95% CI 1.26‐2.60; rank B: HR 2.03, 95% CI 1.31‐3.14; rank C: HR 1.23, 95% CI 0.83‐1.83 vs rank J), emergency department (internal medicine: HR 1.23, 95% CI 0.92‐1.64; emergency department: HR 1.81, 95% CI 1.26‐2.60 vs department of surgery), and history of falls within 1 year (yes: HR 1.66, 95% CI 1.21‐2.27) are shown as predictors of inhospital falls (Table 2, right side).

### Construction of a Fall Scoring System

**Figure 2.**
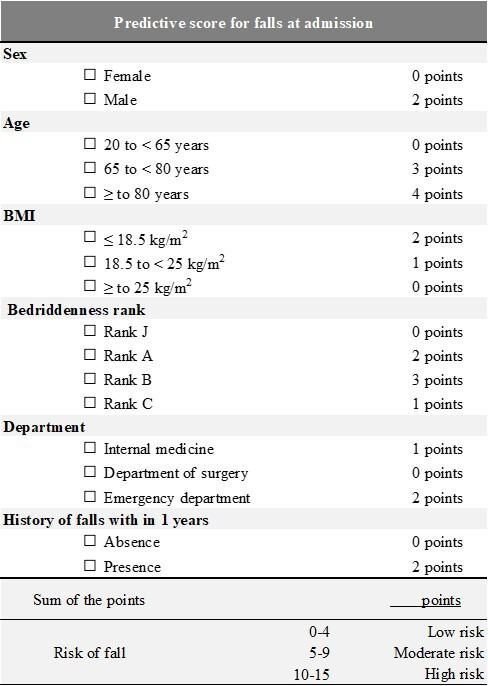
Fall prediction scoring system to be implemented at the time of admission.

**Figure 3.**
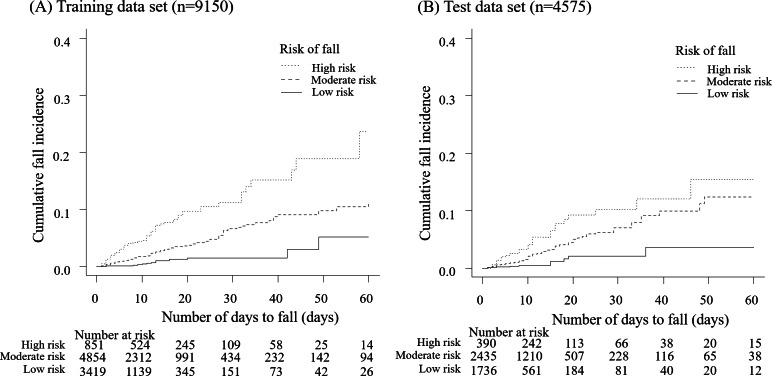
Fall risk classification-specific cumulative fall incidence. Cumulative fall incidences classified by fall risk scoring are shown for the training dataset (**A**) and test dataset (**B**).

Based on the results of multivariable analysis using the training set, we weighted the scores based on each HR (Table 2, right side) and formed 3 fall-risk groups (low risk: 0-4 points, moderate risk: 5-9 points, and high risk: 10-15 points) using conditional inference tree analysis (Figure S1 in Multimedia Appendix 1). The new fall prediction scoring system built on this basis is shown in Figure 2. As a predictor of the performance of these 3 classifications of fall prediction, the c-index in the validation set (n=4561) was 0.733 (95% CI 0.684‐0.782). The cumulative fall incidences in training and test datasets are shown, with Kaplan-Meier curves presented for the training dataset (Figure 3A) and the test dataset (Figure 3B).

## Discussion

### Principal Results

This study was a retrospective cohort investigation conducted at an institution specialized in acute inpatient care, aimed at identifying the risk factors for falls using the time from admission to fall as the outcome variable. Fall risk factors included age, sex, BMI, BR, emergency department, and history of falls within 1 year. Specifically, the study found that older patients (aged 80 years and older) had a higher risk of falls, with men being more at risk than women. Patients with a BMI of <18.5 and those admitted through the emergency department had an increased risk. In addition, those with a history of falls within the past year were particularly vulnerable. We constructed a new predictive scoring system for falls by weighting scores based on each HR according to the results of multivariable analysis and using statistical methods to classify fall risk groups into 3 categories (low risk: 0-4 points, moderate risk: 5-9 points, and high risk: 10-15 points). The present fall prediction scoring system could facilitate preventive interventions for high-risk patients, potentially reducing the likelihood of falls among the most susceptible patient populations and improving patient safety and care in the hospital environment.

### Comparison With Prior Work

In terms of age-specific fall incidence, it was evident that a higher proportion of falls occurred among people aged 65 years and older. This result is in alignment with previous reports [6,16,20,22,27,40] identifying advanced age as a predictive factor for falls. Consistent with past studies [5,16,20,22,40], a history of falls was determined to be a predictive factor.

Previous reports on predictors of falls have shown that sex can be a predictor for both men and women. In this study, being male was identified as a risk factor. In addition, men have been found to experience multiple falls more frequently [27]. Past reports indicating that being male increases the risk of falls [20,28] have focused on hospitalized patients whereas those suggesting an increased risk for women [41,42] have focused on community-dwelling individuals. These differences may be owing to the different characteristics of the study populations, that is, relatively healthy community residents and patients in health care facilities. This may be related to the fact that hospitalized patients tend to have reduced physical activity, which increases the risk of falls. However, the relationship between sex differences and fall risk factors in the hospitalized population remains unclear, and further research is needed to elucidate these aspects.

A systematic review targeting community-dwelling older adults showed that a low BMI (<17 kg/m^2^) is associated with a greater risk of falls, when using 23.5 kg/m^2^ as the baseline [34]. In addition, some reports indicate that both high (25‐35 kg/m^2^ or above) and low (below 18.5 kg/m^2^) BMI values are associated with increased fall risks [31-33,35]. These findings suggest that extreme BMI values influence the risk of falling. However, in our study, we identified low BMI (below 18.5 kg/m^2^) and normal BMI (18.5‐25 kg/m^2^) as risk factors for falls, using high BMI (25 kg/m^2^ or above) as the reference category. This divergence in results might be attributed to the fact that in our Japanese study population, few patients met the international high BMI criterion (>30 kg/m^2^). In the study, because only 5.7% (6/741) of people in this training category fell, BMI >30 kg/m^2^ was not able to be included as the category for a categorical variable BMI.

The BR degree, which is strongly correlated with ADL, was identified as a risk factor for inhospital falls. This finding aligns with several fall prediction models [17,18,20], demonstrating the significant role of a decline in ADL, as it is known that exercise therapy is effective in fall prevention [6,7]. This underscores that impaired ADL is a crucial factor in determining the outcomes of patients who experience falls. In addition, because the overall assessment of ADL is predominantly based on mobility, a substantial correlation [43] between ADL and BR has been observed. In our study, of the 15 variables that showed a correlation with BR, 12 were related to components of ADL.

Being admitted to the emergency department (as an inpatient department) was identified as a risk factor for inhospital falls. The emergency department inpatient population comprises patients who have been transported to the emergency department or who otherwise came voluntarily to the emergency department. This population typically has more severe illness, which may explain why emergency department admission is a risk factor for inhospital falls. Moreover, a history of falls within the past 1 year was identified as a risk factor for inhospital falls. This result was similar to previous reports that identified a fall history as a risk factor for subsequent falls [17,18,20].

Falls are internationally recognized as a serious health issue [3,30], and various efforts to prevent falls are undertaken worldwide. Particularly in Japan, where rapid aging is prevalent, fall prevention has become an increasingly critical issue. Education for patients and staff is considered a fundamental approach to addressing this problem [8,10-12,44], and the introduction and enhancement of educational programs in Japanese hospitals are desirable [10]. To achieve this, it is essential to identify high-priority patients among inpatients and implement fall prevention measures as a high-risk approach. Multifactorial interventions to comprehensively assess and address multiple fall risk factors have proven effective [10]. However, some tools for evaluating fall risk have been criticized for their time-consuming nature and limited effectiveness [45-47], necessitating judicious selection and effective use. Recent research supports these multifactorial interventions and highlights the importance of tailored educational programs and effective risk assessment tools. Guidelines from the United Kingdom’s National Institute for Health and Care Excellence offer specific approaches for fall prevention [48], which could serve as valuable references for fall prevention strategies in Japan. In the approach to fall prevention in Japanese hospitals, international insights and guidelines should be considered while also tailoring unique approaches according to facility characteristics, health care delivery systems, and patient backgrounds.

### Statistical Validation and Clinical Application of Risk Categorization

In our research, we used the conditional inference tree method for statistical analysis to categorize patients into low-, middle-, and high-risk groups, as detailed in Table S4 in Multimedia Appendix 1. This methodological choice ensured that our risk stratifications were based on solid data analysis, avoiding arbitrary determinations. In addition, this scoring approach permits adjustment of cutoff values based on each health care facility’s resources and circumstances, enhancing its applicability in diverse clinical settings. Our aim is not to change the patient’s fall risk level based on resources but to adjust the intensity of preventive interventions according to available resources. The fall risk classification is based solely on clinical characteristics, while resource availability guides the prioritization and distribution of these interventions.

For patients identified as high risk for falls according to our predictive model, it is important to recognize that identifying these individuals is only the first step; providing effective interventions is a separate and critical challenge. Based on prior research demonstrating their effectiveness, we recommend several interventions tailored to implement fall prevention measures as a high priority. Here, we outline interventions such as increased monitoring, personalized environmental adjustments, nonslip footwear, assistive devices such as walkers or canes, one-on-one support, and immediate assistance. In addition, patient education and rehabilitation are crucial components. Educating both patients and health care providers about fall risks and preventive strategies, combined with physical therapy to enhance strength and balance, can significantly reduce the risk of falls [6-12]. However, it may be necessary to adjust these cutoffs based on sensitivity and specificity considerations to enhance the accuracy of patient risk identification, aligning more closely with practical prediction practices in health care.

The fall prediction tool we developed integrates seamlessly with a hospital’s EHR system. Upon a patient’s admission, it automatically retrieves EHR data and calculates a fall risk score based on various predictors. This integration allows all health care providers, including emergency and trauma physicians, to easily access the patient’s fall risk assessment. Emergency or trauma physicians can use this tool to quickly identify patients at high risk for falls and implement appropriate interventions. This proactive approach can significantly reduce the incidence of falls and enhance patient safety in fast-paced and high-stress environments such as emergency departments and trauma centers.

### Limitations

This study has several limitations. First, owing to the identification of inhospital fall predictors based on the characteristics of the facility and patient population in an acute care hospital, generalizing the findings to other all hospitals may be challenging. While the generalizability of our model is not fully guaranteed due to the lack of external validation, hospitals with similar facilities and patient characteristics might find the identified predictors and scoring system developed in this study applicable to their setting. Second, our study did not examine psychiatric symptoms, including delirium, as a potential predictive factor [49,50]. Because previous studies have shown that psychiatric symptoms can be a risk factor for falls [17,18,28], it can be necessary to reconstruct the prediction model by including additional predictors of falls, including psychiatric symptoms such as delirium, in future studies. Third, we did not examine the association between medications and falls. Previous studies have shown that taking sleeping pills [19,20,28-30,51] and the number of medications [42,52-54] are risk factors for falls. At the time of our study, limitations in our EHR system made it difficult to collect accurate medication data. This prevented us from including medication type and number as predictors. Since then, our EHR system has been upgraded, and we can now reliably obtain medication data. We plan to incorporate these variables in future studies to enhance the accuracy of our fall prediction model. Fourth, we could not compare the results of our model with those of existing fall prediction models. However, we believe that our newly developed fall prediction model is highly useful in that it is easy to apply in many Japanese hospitals with acute care settings, similar to that in this study.

To further enhance the practical application of our fall prediction scoring system, we plan to integrate it into our hospital’s EHRs to gather real-world evidence. This will allow us to evaluate its usability, accuracy, and impact on reducing inhospital falls. Future research will include multisite longitudinal studies to validate the tool’s effectiveness across different health care settings.

### Conclusions

We successfully identified predictors of falls within a patient population admitted to an acute care hospital and developed a novel prediction model in Japan. This model could serve as an effective tool to guide preventive interventions for both individual hospitalized patients and high-risk populations in many hospitals with acute care settings.

## Supplementary material

10.2196/58073 Multimedia Appendix 1 Supplementary tables and figure.

## Abbreviations

ADL: activities of daily living
BR: bedriddenness rank
EHR: electronic health record
HR: hazard ratio
