# Supplementary material for: Predictive Factors and the Predictive Scoring System for Falls in Acute Care Inpatients: Retrospective Cohort Study
Source: JMIR Hum Factors. 2025 Jan 13;12:e58073. doi: 10.2196/58073 (PMC11897365; doi:10.2196/58073)
Supplement: Multimedia Appendix 1 [file humanfactors-v12-e58073-s001.docx]

**Figure S1.** Results of conditional inference tree analysis to identify fall risk categories.


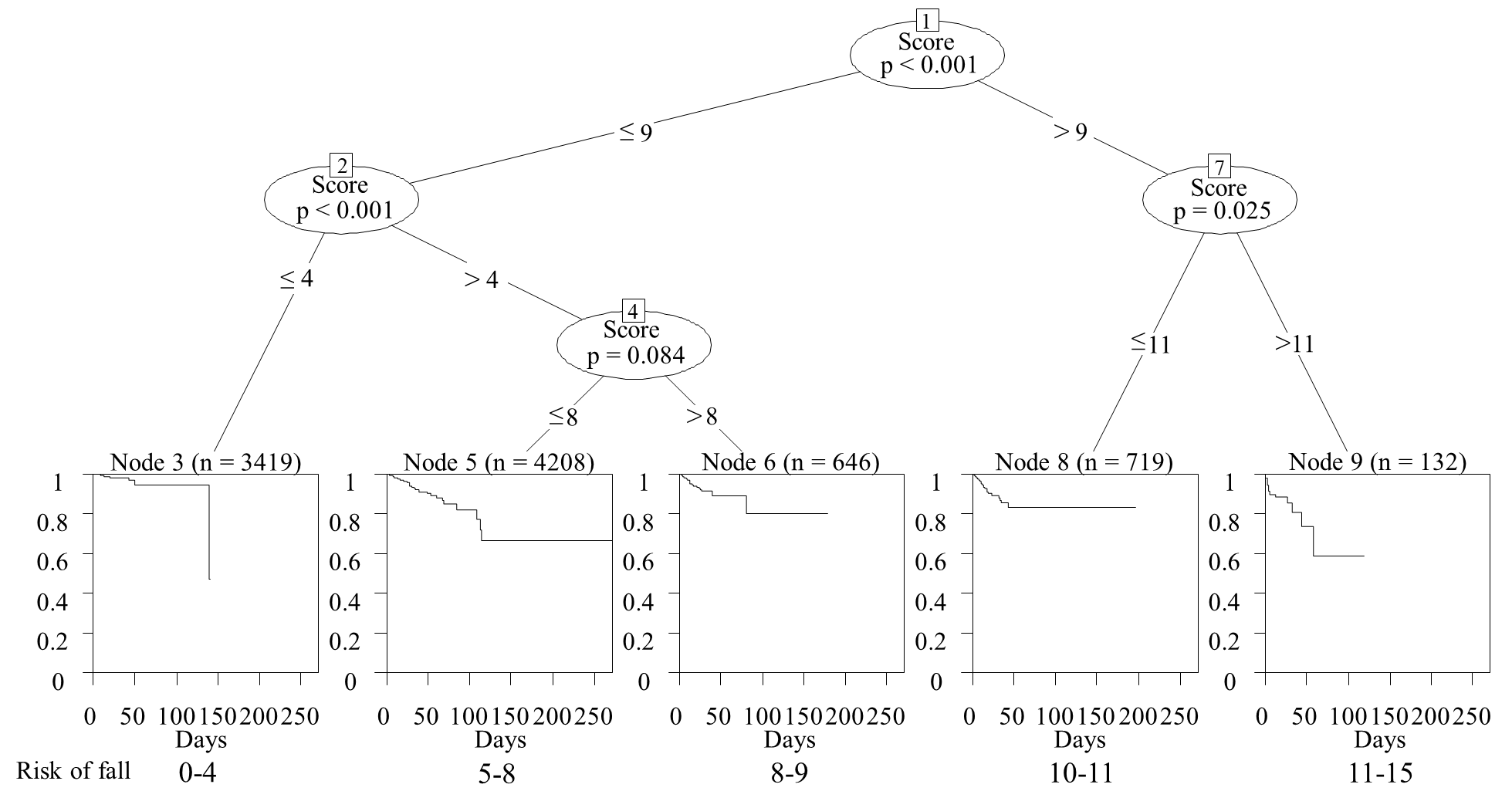


**Table S1.** The classification of fall incident or accident levels by the Medical Safety Management Council of National University Hospitals in Japan (2002).

| **Level** | **Outcome** | **Severity** | **Contents** |
| --- | --- | --- | --- |
| Level 0 | ー | ー | Errors and drug/medical device malfunctions were observed but not implemented on the patient |
| Level 1 | None | ー | No real harm to the patient (Can't rule out the possibility that it had some impact) |
| Level 2 | Transient | Mild | No procedure or treatment (The need for increased observation, mild changes in vital signs, and tests to confirm safety arose) |
| Level 3a | Transient | Moderate | It required a simple procedure or treatment (Disinfecting, Poultice, suturing the skin, administering painkillers, etc.) |
| Level 3b | Transient | Severe | It required intense treatment and procedures (Severe changes in vital signs, placement on ventilator, surgery, extended hospital stay, Hospitalization of outpatients, fracture, etc.) |
| Level 4a | Permanent | Mild to moderate | Permanent disability or sequelae, but not accompanied by significant functional impairment or cosmetic problems |
| Level 4b | Permanent | Moderate to severe | Permanent disability or residual disability, but with significant functional impairment or cosmetic problems |
| Level 5 | Death |  | Death (Excluding those caused by the natural course of the primary disease) |

**Table S2.** Comparison between Patients backgrounds in training and test datasets.

| Variable | Category | Training data set (n = 9,105) |  | Test set (n = 4,575) | *P-*value |
| --- | --- | --- | --- | --- | --- |
|  |  |  |  |  |  |
|  |  |  |  |  |  |
|  |  | Number (%) |  | Number (%) |  |
| Presence or absence of a fall | Without fall | 8,934 (97.6) |  | 4,465 (97.6) | 0.921 |
|  | With fall | 216 (2.4) |  | 110 (2.4) |  |
| Age | 20 to <65 years | 3,375 (36.9) |  | 1,747 (38.2) | 0.227 |
|  | 65 to <80 years | 3,778 (41.3) |  | 1,877 (41.0) |  |
|  | ≥to 80 years | 1,997 (21.8) |  | 951 (20.8) |  |
| Sex | Female | 4,362 (47.7) |  | 2,213 (48.4) | 0.450 |
|  | Male | 4,788 (52.3) |  | 2,362 (51.6) |  |
| Body mass index | <to 18.5 kg/m^2^ | 1,190 (13.0) |  | 606 (13.2) | 0.899 |
|  | 18.5 to <25 kg/m^2^ | 5,495 (60.1) |  | 2,761 (60.3) |  |
|  | ≥to 25 kg/m^2^ | 2,439 (26.7) |  | 1,194 (26.1) |  |
|  | Missing | 26 (0.3) |  | 14 (0.3) |  |
| Dementia | No | 8,908 (97.4) |  | 4,440 (97.0) | 0.328 |
|  | Yes | 242 (2.6) |  | 135 (3.0) |  |
| Parkinson disease | No | 9,074 (99.2) |  | 4,532 (99.1) | 0.580 |
|  | Yes | 76 (0.8) |  | 43 (0.9) |  |
| Stroke | No | 8,304 (90.8) |  | 4,156 (90.8) | 0.892 |
|  | Yes | 846 (9.2) |  | 419 (9.2) |  |
| Visual impairment | No | 8,186 (89.5) |  | 4,095 (89.5) | 0.961 |
|  | Yes | 964 (10.5) |  | 480 (10.5) |  |
| Cognitive function scores | No | 8,034 (87.8) |  | 4,043 (88.4) | 0.182 |
|  | Yes | 747 (8.2) |  | 335 (7.3) |  |
|  | Missing | 369 (4.0) |  | 197 (4.3) |  |
| Ambulance transport | No | 5,602 (61.2) |  | 2,824 (61.7) | 0.581 |
|  | Yes | 3,548 (38.8) |  | 1,751 (38.3) |  |
| Emergency admission | Scheduled hospitalization | 7,588 (82.9) |  | 3,839 (83.9) | 0.153 |
|  | Emergency hospitalization | 1,562(17.1) |  | 736 (16.1) |  |
| Department | Internal medicine | 4,614 (50.4) |  | 2,316 (50.6) | 0.974 |
|  | Department of Surgery | 4,105 (44.9) |  | 2,043 (44.7) |  |
|  | Emergency Department | 431 (4.7) |  | 216 (4.7) |  |
| Consciousness disorders | No | 8,189 (89.5) |  | 4,122 (90.1) | 0.288 |
|  | Yes | 961 (10.5) |  | 453 (9.9) |  |
| Requirement for nursing care | No | 1,602 (17.5) |  | 810 (17.7) | 0.582 |
|  | Yes | 1,373 (15.0) |  | 656 (14.39 |  |
|  | Missing | 6,175 (67.5) |  | 3,109 (68.0) |  |
| Bedriddenness rank | Rank J | 5,565 (60.8) |  | 2,787 (60.9) | 0.715 |
|  | Rank A | 1,626 (17.8) |  | 806 (17.6) |  |
|  | Rank B | 560 (6.1) |  | 261 (5.7) |  |
|  | Rank C | 1,399 (15.3) |  | 721 (15.8) |  |
| Good sleep condition | Good | 5,040 (55.1) |  | 2,502 (54.7) | 0.535 |
|  | Bad | 1,156 (12.6) |  | 609 (13.3) |  |
|  | Missing | 2,954 (32.3) |  | 1,464 (32.0) |  |
| Use of sleeping medication | No | 4,446 (48.6) |  | 2,280 (49.8) | 0.229 |
|  | Yes | 924 (10.1) |  | 427 (9.3) |  |
|  | Missing | 3,780 (41.3) |  | 1,868 (40.8) |  |
| Status of medication management | Myself | 3,489 (38.1) |  | 1,749 (38.2) | 0.762 |
|  | Others | 404 (4.4) |  | 214 (4.7) |  |
|  | Missing | 5,257 (57.5) |  | 2,612 (57.1) |  |
| Eating | Independent | 7,174 (78.4) |  | 3,591 (78.5) | 0.924 |
|  | Requiring assistance | 1,976 (21.6) |  | 984 (21.5) |  |
| Transferring | Independent | 6,668 (72.9) |  | 3,339 (73.0) | 0.908 |
|  | Requiring assistance | 2,482 (27.1) |  | 1,236 (27.0) |  |
| Dressing | Independent | 7,177 (78.4) |  | 3,601 (78.7) | 0.786 |
|  | Requiring assistance | 1,957 (21.4) |  | 964 (21.1) |  |
|  | Missing | 16 (0.2) |  | 10 (0.2) |  |
| Toilet transfer/use | Independent | 6,914 (75.6) |  | 3,470 (75.8) | 0.509 |
|  | Requiring assistance | 2,220 (24.3) |  | 1,093 (23.9) |  |
|  | Missing | 16 (0.2) |  | 12 (0.3) |  |
| Bathing | Independent | 6,768(74.0) |  | 3,388 (74.1) | 0.138 |
|  | Requiring assistance | 2,156 (23.6) |  | 1,049 (22.9) |  |
|  | Missing | 226 (2.5) |  | 138 (3.0) |  |
| Level walking | Independent | 6,800 (74.3) |  | 3,402 (74.4) | 0.477 |
|  | Requiring assistance | 2,229 (24.4) |  | 1,101 (24.1) |  |
|  | Missing | 121 (1.3) |  | 72 (1.6) |  |
| Stair use | Independent | 6,627 (72.4) |  | 3,309 (72.3) | 0.469 |
|  | Requiring assistance | 2,097 (22.9) |  | 1,032 (22.6) |  |
|  | Missing | 426 (4.7) |  | 234 (5.1) |  |
| Changing clothes | Independent | 6,896 (75.4) |  | 3,472 (75.9) | 0.783 |
|  | Requiring assistance | 2,235 (24.4) |  | 1,093 (23.9) |  |
|  | Missing | 19 (0.2) |  | 10 (0.2) |  |
| Defecation management | Independent | 7,381 (80.7) |  | 3,693 (80.7) | 0.902 |
|  | Requiring assistance | 1,705 (18.6) |  | 847 (18.5) |  |
|  | Missing | 64 (0.7) |  | 35 (0.8) |  |
| Urinary management | Independent | 7,369 (80.5) |  | 3,685 (80.5) | 1.000 |
|  | Requiring assistance | 1,713 (18.7) |  | 856 (18.7) |  |
|  | Missing | 68 (0.7) |  | 34 (0.7) |  |
| History of falls within 1 year | No | 7,983 (87.2) |  | 3,991 (87.2) | 1.000 |
|  | Yes | 1,167 (12.8) |  | 584 (12.8) |  |
| Inability to stand without holding | No | 5,371 (58.7) |  | 2,667 (58.3) | 0.878 |
|  | Yes | 3,645 (39.8) |  | 1,838 (40.2) |  |
|  | Missing | 134 (1.5) |  | 70 (1.5) |  |
| Impaired judgment and comprehension | No | 7,749 (84.7) |  | 3,878 (84.8) | 0.522 |
|  | Yes | 1,169 (12.8) |  | 595 (13.0) |  |
|  | Missing | 232 (2.5) |  | 102 (2.2) |  |
| Toileting assistance | No | 6,800 (74.3) |  | 3,392 (74.1) | 0.765 |
|  | Yes | 1,894 (20.7) |  | 965 (21.1) |  |
|  | Missing | 456 (5.0) |  | 218 (4.8) |  |
| Use of portable toilet | No | 7,815 (85.4) |  | 3,954 (86.4) | 0.249 |
|  | Yes | 459 (5.0) |  | 220 (4.8) |  |
|  | Missing | 876 (9.6) |  | 401 (8.8) |  |
| Physical restraint screening | No | 6,665 (72.8) |  | 3,345(73.1) | 0.943 |
|  | Yes | 278 (3.0) |  | 137 (3.0) |  |
|  | Missing | 2,207 (24.1) |  | 1,093 (23.9) |  |

Categorical variables are presented as the number of patients and percentages. BMI, body mass index; Between-group comparisons were made using t-tests and chi-square tests for continuous and categorical variables, respectively. The P-value is from the Wald test. Bedriddenness rank J means independent/autonomous, A means housebound, B means chair and C means bedriddenness; HR, odds ratio; CI, confidence interval. Between-group comparisons were made using t-tests and chi-square tests for continuous and categorical variables, respectively. P-value for Wald test.

**Table S3.** Frequency of falls for all incident levels in an entire population (n=13,725).

| **Level** | **Number of patents with fall (489 cases)** |
| --- | --- |
| Level 0 | 1 |
| Level 1 | 162 |
| Level 2 | 277 |
| Level 3a | 40 |
| Level 3b | 8 |
| Level 4a | 1 |
| Level 4b | 0 |
| Level 5 | 0 |

**Table S4. Spearman’s rank correlation coefficients for predictors in the training dataset.**

|  | Age | Sex | BMI | Emergency admission | Bedriddenness rank | Department | consciousness disorders | Eating | Transferring | Dressing | Toilet transfer/use | Bathing | Level walking | Stair use | Changing clothes | Defecation management | Urination management | History of falls within 1 year | Inability to stand without holding | Impaired judgment and comprehension | Toileting assistance |
| --- | --- | --- | --- | --- | --- | --- | --- | --- | --- | --- | --- | --- | --- | --- | --- | --- | --- | --- | --- | --- | --- |
| Age | 1 |  |  |  |  |  |  |  |  |  |  |  |  |  |  |  |  |  |  |  |  |
| Sex | 0.088 | 1 |  |  |  |  |  |  |  |  |  |  |  |  |  |  |  |  |  |  |  |
| BMI | -0.152 | 0.056 | 1 |  |  |  |  |  |  |  |  |  |  |  |  |  |  |  |  |  |  |
| Emergency admission | 0.168 | 0.005 | -0.131 | 1 |  |  |  |  |  |  |  |  |  |  |  |  |  |  |  |  |  |
| Bedriddenness rank | 0.324 | 0.01 | -0.151 | **0.6** | 1 |  |  |  |  |  |  |  |  |  |  |  |  |  |  |  |  |
| Department | 0.002 | 0.009 | 0.011 | -0.115 | 0.014 | 1 |  |  |  |  |  |  |  |  |  |  |  |  |  |  |  |
| consciousness disorders | 0.257 | -0.006 | -0.145 | 0.349 | **0.468** | 0.039 | 1 |  |  |  |  |  |  |  |  |  |  |  |  |  |  |
| Eating | 0.329 | 0.006 | -0.17 | **0.537** | **0.701** | 0.009 | **0.563** | 1 |  |  |  |  |  |  |  |  |  |  |  |  |  |
| Transferring | 0.348 | 0.009 | -0.156 | **0.588** | **0.725** | -0.013 | **0.499** | **0.841** | 1 |  |  |  |  |  |  |  |  |  |  |  |  |
| Dressing | 0.329 | 0.013 | -0.163 | **0.537** | **0.7** | 0.012 | **0.552** | **0.907** | **0.843** | 1 |  |  |  |  |  |  |  |  |  |  |  |
| Toilet transfer/use | 0.351 | 0.011 | -0.168 | **0.568** | **0.721** | -0.003 | **0.532** | **0.885** | **0.907** | **0.89** | 1 |  |  |  |  |  |  |  |  |  |  |
| Bathing | 0.354 | 0.009 | -0.164 | **0.566** | **0.719** | -0.004 | **0.504** | **0.852** | **0.892** | **0.861** | **0.914** | 1 |  |  |  |  |  |  |  |  |  |
| Level walking | 0.349 | 0.007 | -0.167 | **0.567** | **0.722** | -0.007 | **0.513** | **0.857** | **0.911** | **0.855** | **0.916** | **0.912** | 1 |  |  |  |  |  |  |  |  |
| Stair use | 0.357 | 0.005 | -0.16 | **0.555** | **0.708** | -0.018 | **0.479** | **0.804** | **0.878** | **0.803** | **0.874** | **0.915** | **0.919** | 1 |  |  |  |  |  |  |  |
| Changing clothes | 0.345 | 0.009 | -0.162 | **0.563** | **0.719** | 0 | **0.529** | **0.886** | **0.896** | **0.895** | **0.93** | **0.917** | **0.91** | **0.879** | 1 |  |  |  |  |  |  |
| Defecation management | 0.321 | 0.003 | -0.158 | **0.522** | **0.677** | 0.022 | **0.585** | **0.866** | **0.792** | **0.864** | **0.849** | **0.804** | **0.816** | **0.759** | **0.843** | 1 |  |  |  |  |  |
| Urination management | 0.324 | 0.004 | -0.156 | **0.519** | **0.677** | 0.018 | **0.585** | **0.865** | **0.792** | **0.867** | **0.849** | **0.805** | **0.815** | **0.759** | **0.845** | **0.984** | 1 |  |  |  |  |
| History of falls within 1 year | 0.193 | -0.042 | -0.057 | 0.189 | 0.28 | 0.121 | 0.243 | 0.296 | 0.299 | 0.297 | 0.3 | 0.303 | 0.3 | 0.308 | 0.306 | 0.283 | 0.285 | 1 |  |  |  |
| Inability to stand without holding | 0.4 | -0.01 | -0.132 | **0.487** | **0.613** | 0.008 | 0.341 | **0.536** | **0.611** | **0.54** | **0.585** | **0.595** | **0.594** | **0.61** | **0.576** | **0.504** | **0.506** | 0.305 | 1 |  |  |
| Impaired judgment and comprehension | 0.261 | -0.012 | -0.142 | 0.293 | **0.407** | 0.024 | **0.556** | **0.477** | **0.437** | **0.47** | **0.464** | **0.445** | **0.447** | **0.427** | **0.457** | **0.486** | **0.487** | 0.272 | **0.419** | 1 |  |
| Toileting assistance | 0.262 | 0.021 | -0.115 | **0.425** | **0.518** | -0.002 | 0.356 | **0.538** | **0.551** | **0.543** | **0.58** | **0.559** | **0.562** | **0.541** | **0.562** | **0.531** | **0.533** | 0.288 | **0.529** | **0.463** | 1 |

Bold type indicates a correlation of > 0.4. BMI, body mass index.
